# Analysis of the advantages of 3D printing in the surgical treatment of multiple rib fractures: 5 cases report

**DOI:** 10.1186/s13019-019-0930-y

**Published:** 2019-06-11

**Authors:** Xue-tao Zhou, Dong-sheng Zhang, Yang Yang, Guo-liang Zhang, Ze-xin Xie, Meng-hui Chen, Zheng Liang

**Affiliations:** Department of Cardiothoracic Surgery, Shijiazhuang Third Hospital, No.15 Tiyu South Street, Chang’an District, Shijiazhuang, 050000 Hebei Province China

**Keywords:** 3D printing technology, Locking plate, Multiple rib fractures, Open reduction and internal fixation

## Abstract

**Background:**

Rib fractures account for a fairly high proportion of chest injuries, ranging from 55 to 80%. The most common mechanisms of injury include: traffic accident, extrusion and falls from significant heights. Besides, the surgical treatment of multiple rib fractures has been accepted by more and more medical professionals. We reported 5 clinical cases of patients with multiple rib fractures undergoing open reduction and internal fixation using 3D printing technology.

**Case presentation:**

Retrospective analysis of 5 clinical cases of multiple rib fractures from January 2017 to August 2018 in our hospital. A preoperative CT thin slice scan was used to reconstruct the 3D model according to the scanning results, and 3D printing technology was adopted to prepare the rib model. Preoperative reconstruction of the rib’s normal shape and lock plate for the shaped ribs was created according to reconstructed model. For multiple fractures especially patients with severely deformed rib shape, it is suggested to intraoperative shape directly to the metal bone plate fixed on the ribs on both ends of the fracture line, in order to establish a basic support frame. The other various fracture section can be fixed on the lock plate respectively. Postoperative chest radiographs of the 5 patients showed that the internal fixations were in good and natural shape. The thoracic contour was well formed and symmetrically with the contralateral side.

**Conclusion:**

Making the rib model and the pre-shaped titanium alloy rib locking plate using 3D printing technology, provided a more minimally invasive and precisely individualized treatment for some rib fracture operations.

## Background

Rib fractures account for a fairly high proportion of chest injuries, ranging from 55 to 80% [1]. In recent years, a large number of domestic and foreign studies have shown that surgical intervention for multiple rib fractures is more effective [2]. In addition, the indications for internal fixation of rib fractures are constantly updated and widened [3], but not include certain types of rib fractures, such as long segment, crushed rib fracture; and special site fractures, such as: 1) pectoralis major muscle coverage, especially in women with breast coverage, 2) scapula coverage area, 3) rib arch site, etc. How to choose the surgical incision site and reduce significant tissue damage are the issues we should consider. However, the reduction of rib fracture and good reconstruction of thoracic cavity are undoubtedly the key to the success of operation. This requires us to be as targeted as possible.

Three-dimensional printing technology has matured in multiple medical fields, among which its advantages have been highlighted in fracture treatment [4]. 3D printing technology enables rapid construction of accurate, complete individual fracture models so that surgeons can perform surgery with models. In this study, we reported on our experience using of 3D printing technology for the treatment of multiple rib fractures cases.

The cardiothoracic surgery department of our hospital treated a total of 5 patients with multiple rib fractures of the above types from January 2017 to August 2018. 3D models of the rib fractures were reconstructed and printed according to the results of thin-layer CT scans before surgeries. The titanium alloy rib locking plate was accurately shaped according to the model, which significantly facilitated the operation procedure.

## Case presentation

### Model production by 3D printing and operative technique

There were 5 patients in this group. The indications of all the five patients were multiple rib fractures, most of which had obvious dislocations of the broken ends and chest walls deformity. All patients had multi-row CT scans and were diagnosed with multiple rib fractures. A summary of the patients’ general demographics was shown in Table 1. These 5 patients were given active treatment after admission, and the surgeries were performed when patients were in stable general conditions, without emergency surgeries. Before the surgeries, all the above types of rib fractures were treated with 3D printing technology to make the rib model and pre-formed a titanium alloy rib locking plate.

**Table 1:** Patient Demographic Information

Case	Gender	Age	Cause of injury	The condition of rib fractures	Associated injury				Operative time of trauma
					Haemothorax	Pneumothorax	Pulmonary complications	Extrathoracic injury	
1	Male	61	Extrusion	Left side 1–6 rib fractures, 3, 4 anterior multiple fractures	Yes	Yes	Pulmonary contusion	No	3
2	Male	57	Fall	Left side 2–12 rib fractures, 4, 5 costal cartilage, and 6 rib anterior costal arch fracture	No	No	Pulmonary contusion	Subarachnoid hemorrhage	8
3	Female	64	traffic accident	The left side is 2–11, where 2–6 contains the costal cartilage multiple fractures involving the costal arch	Yes	No	Yes	No	5
4	Male	54	traffic accident	Left side 3–6 anterior multiple fractures involving the cartilage	Yes	Yes	Yes	Clavicular fracture	4
5	Male	56	traffic accident	Right side 2–6 anterior multiple fractures, 5–10 lateral posterior ribs	Yes	No	Yes	Clavicular fracture, Subarachnoid hemorrhage	7

### Case report one

**Fig. 1:**
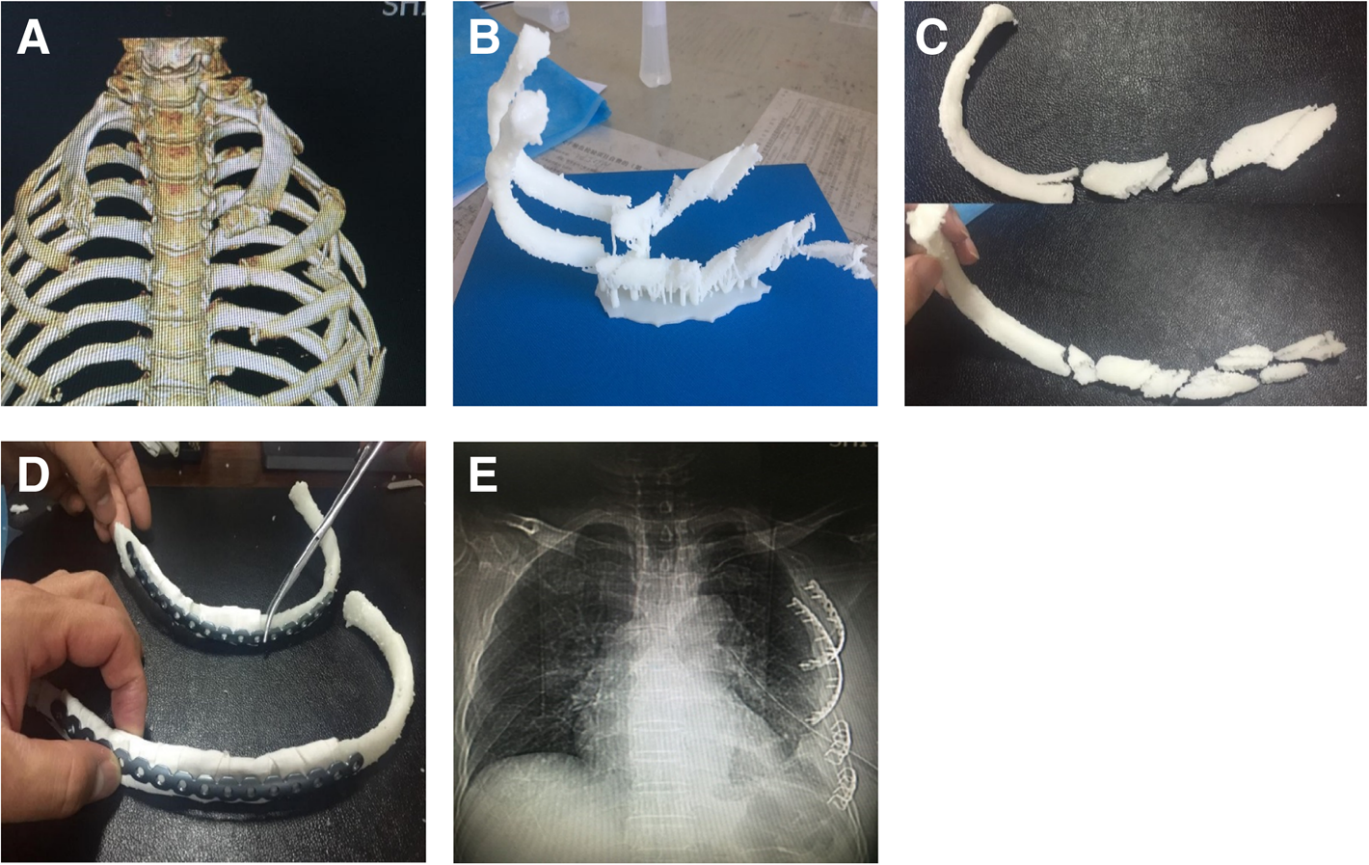
No.1 patient (**a**) Preoperative scanning revealed long segment comminuted fractures in 3 and 4 ribs. **b** Preoperative 3 and 4 rib models were prepared by 3D printing technology based on CT thin slice scanning. **c**, **d** Reduction fracture morphology; the 3D printing model was spliced and the titanium alloy rib locking plate was shaped according to the mode. **e** Postoperative review, the shape of the internal fixator was intact, and the shape of the contralateral rib was perfectly symmetrical compared with preoperative image A

We reported the case of a 61-year-old male who suffered an extrusion of intercostals nerve with 1–6 left rib fractures among which ribs 3 and 4 were long comminuted fractures (see Fig. 1a). It was proposed to perform open reduction and internal fixation surgery on ribs 3–6. In view of long segment comminuted fractures of ribs 3 and 4 at a relatively high position, with pectoral muscle covering in front and scapula covering in the rear, reduction and fixation of this two-rib fracture was the key to a successful surgery.

A preoperative CT thin slice scan was used to reconstruct the 3D model according to the scanning results, and the models of ribs 3 and 4 were prepared using 3D printing (Fig. 1b). The 3D printed model of each fracture segment of the two ribs was adhered and reconstructed respectively (Fig. 1c). The two-rib titanium alloy frame locking plate was respectively shaped according to the reconstructed model (Fig. 1d).

The patient was treated with general anesthesia, right lateral position, and a 8-cm incision was made under the lower edge of the 4th rib. The skin and the subcutaneous tissue were separated layer by layer, revealing the anterior latissimus dorsi and musculus serratus anterior. A tunnel-type operating space was made by disconnecting from the back of the pectoralis major and the pectoralis minor to the rear of the scapula along the of surface 3rd and 4th ribs. Under the assistance of endoscope, the titanium alloy rib locking plate, which was shaped before surgery, was placed on the 3rd rib’s surface, and was well fitted with the non-fractured end of the 3rd rib front and rear. The long-angled clamp temporarily affixed the metal internal fixation plate to the rib, and used the Matrix RIB: MIPO system to drill holes. Then, two screws were inserted into both ends and locked, thus the metal internal fixation plate was firmly affixed. A plurality of small fracture segments in the middle were respectively drilled and fixed onto the locking plate. The 4th rib was fixed in the same way. The chest wall was well shaped after surgery (Fig. 1e).

### Case report two

**Fig. 2:**
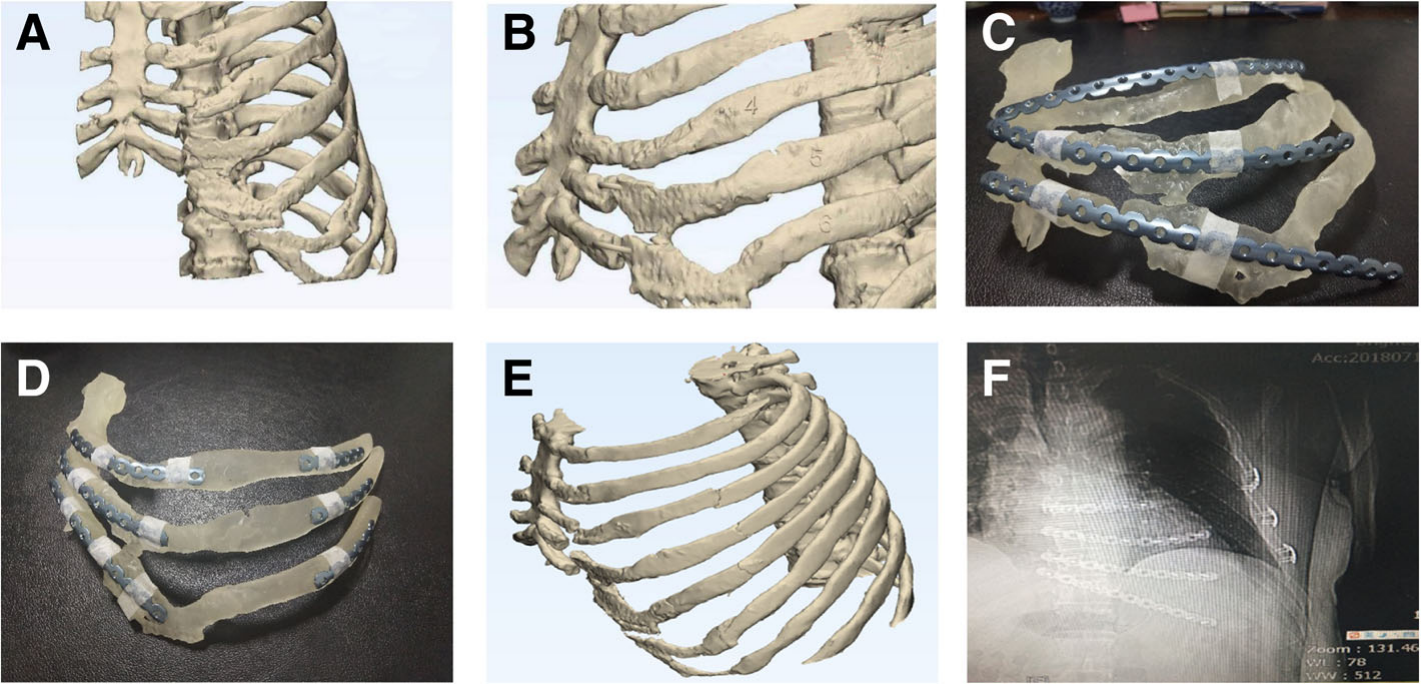
No.2 patient (**a**) According to the 3D model made by CT, 4 and 5 costal cartilage fractures and 6 costal anterior costal arch fracture can be seen. **b** The 3D model of the fracture end of the rib was adjusted by using 3D software model. **c** The 3D printed 4–6 rib model shows a large gap between the locking plate and the shape of the rib. **d** After giving the shape, the locking plate and the rib paste are in good condition. **e**, **f** Postoperative review, the shape of the internal fixator was intact, and the shape of the contralateral rib recovered well compared with the preoperative image

The second case was a 57-year-old male with multiple fractures of the left ribs, including 4 and 5 costal cartilage and rib 6 anterior costal arch fractures (Fig. 2a). Because this part was cartilage, including part of the costal arch, and the ribs were not in regular shape, the fixation firmness of costal cartilage was not as good as that of common bone. Therefore, it was proposed that the inner end of the locking plate should be affixed to the sternum and the outer end to the rib bone.

The preoperative CT thin slice scan was used to reconstruct the 3D model according to the scanning results, and the fractured end of the rib fractures were adjusted and restored using software (Fig. 2b). The 3 and 4 rib models were prepared using 3D printing, and the titanium alloy rib locking plate was prefabricated accordingly (Fig. 2c, d).

During the operation, the 5th rib oblique incision was taken as the center of the rib fracture according to CT and palpation of fracture end, separated layer by layer. Attention should be paid to the protection of the muscular layer, and the muscle fiber was split to expose the broken end of the rib for drilling, without excessive dissection. Then the inner end of the 4th and 5th ribs were affixed to the sternum, and the distal end was affixed to the rib bone part, and the two ends of the middle cartilage were respectively affixed by 1 or 2 screws. The costal arch can only be affixed by drilling into the costal cartilage due to anatomical limitations. Chest wall was well formed after operation (Fig. 2e, f).

### Case report three

**Fig. 3:**
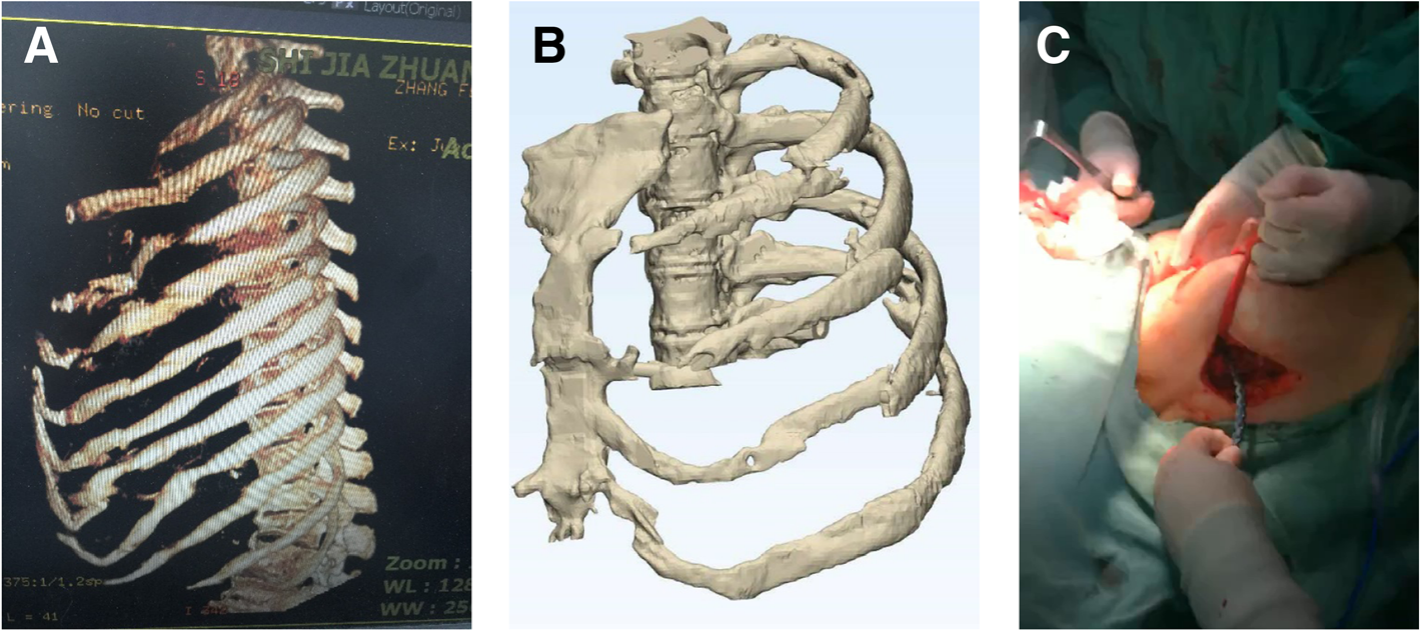
No.3 patient (**a**) Preoperative scanning revealed 2–11 left rib fractures where 2–6 ribs contained the costal cartilage multiple fractures involving the costal arch. **b** Preoperative 3–5 rib models were prepared by 3D printing technology based on CT thin slice scanning. **c** During the surgery, corresponding pre-shaping locking plates were placed in each rib

A 64-year-old female was admitted to our hospital because of traffic accident with 2–11 left rib fractures where 2–6 contained the costal cartilage multiple fractures involving the costal arch (Fig. 3a). Because the No.3 patient was a female, the operation should not only consider minimally invasive, but also need to protect breast tissue adequately. Moreover, considering the stability of the fixator, the medial side of the locking plate was fixed in the body of the sternum, and then the sternum and armpit were treated with tunneling open reduction and internal fixation.

A preoperative CT thin slice scan was used to reconstruct the 3D model according to the scanning results, and the models of ribs 3–5 were prepared using 3D printing (Fig. 3b). The titanium alloy frame locking plate was re-shaped according to the reconstructed model.

Intraoperatively, a vertical incision (4 cm long) was performed on the body part of the sternum in the patient, which was free to the bone surface. An 8-cm vertical incision below the armpit was separated layer by layer. Attention should be paid to protect the muscular layer, and the muscle gap or the muscle fiber was split to expose the broken end of the rib, avoiding traversing muscle tissue. Next, the surface of the rib loose tissue was split to the side of the sternum, merging with the chest incision. Then, the broken ends of each rib were slightly split, and the broken ends of each fracture were gently repositioned. Corresponding pre-shaping locking plates were placed in each rib (Fig. 3c). The proximal sternum was drilled and fixed with two screws; the other end was fixed on the distal bone part of the fracture line with two screws; and the middle cartilage was fixed with 1 to 2 screws at each end. The surgery was completed successfully.

### Case report four and five

**Fig. 4:**
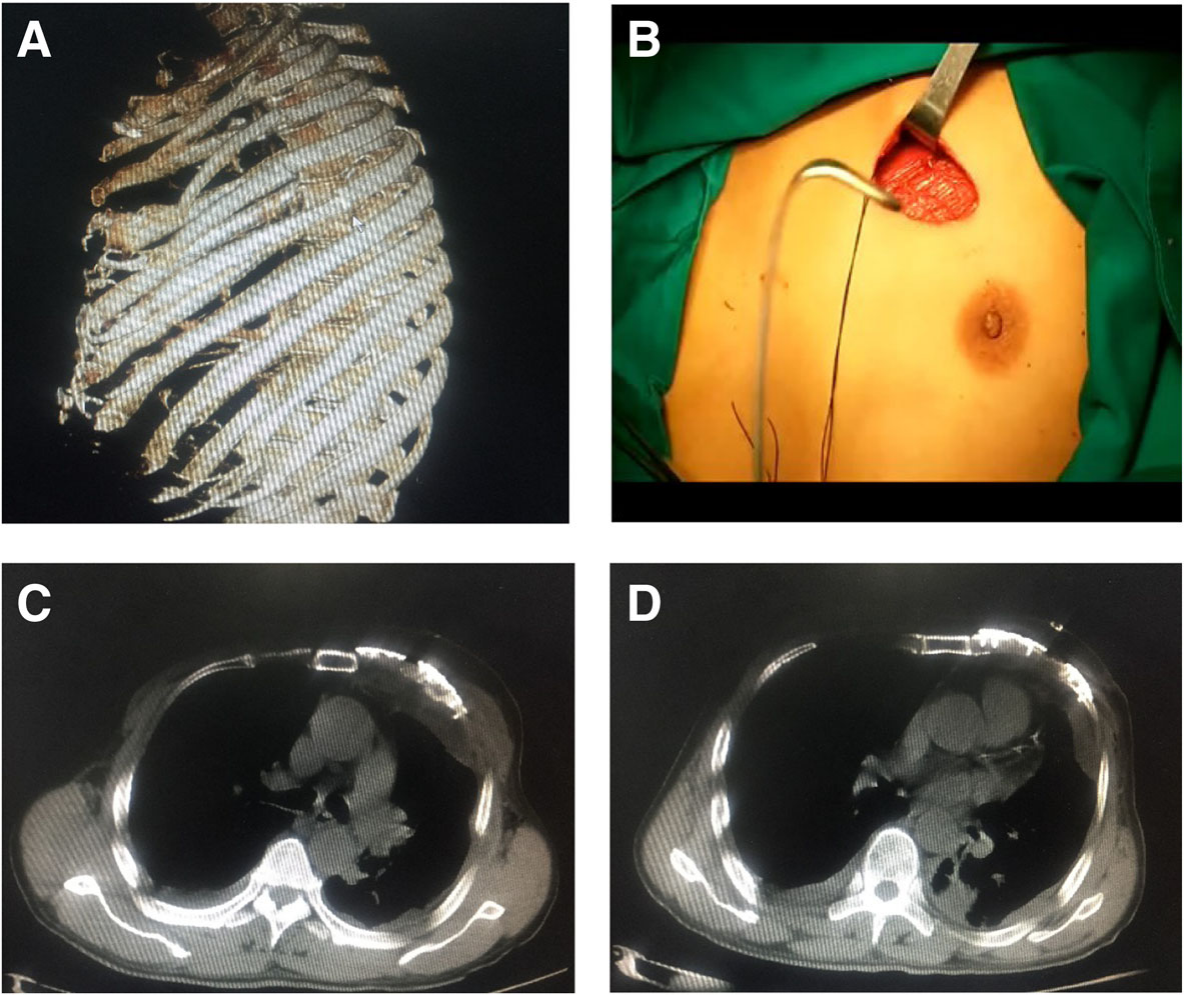
No.4 patient (**a**) Preoperative scanning revealed 3–6 anterior multiple fractures in the left side involving the cartilage. **b** During the surgery, a 6-cm chest incision was made. **c**, **d** The postoperative CT locking plate position and thoracic morphology were satisfactory

The other two male patients all suffered multiple rib fractures caused by the car accident; the No. 4 patient had 3–6 anterior multiple fractures in the left side involving the cartilage (Fig. 4a); the No. 5 patient had 2–6 anterior multiple fractures in the right side, and 5–10 lateral posterior ribs. Because these 2 patients were male without the influence of breast tissue, and the surgical path and surgical method are similar by performing tunneling open reduction and internal fixation.

**Fig. 5:**
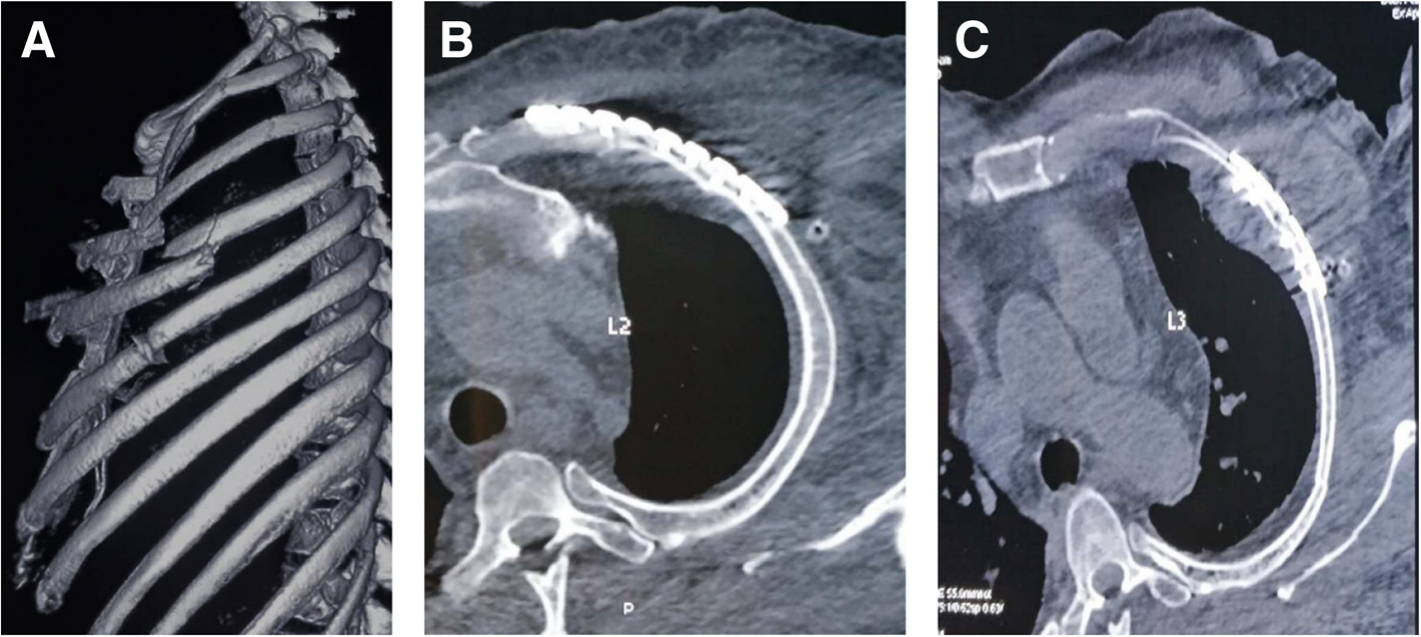
No.5 patient (**a**) The 3D model of the fracture end of the rib was adjusted by using 3D software model. **b**, **c** The postoperative CT locking plate position and thoracic morphology were great

The preoperative CT thin layer scan was used to reconstruct the 3D model according to the scanning results, and the fractured end of the ribs were adjusted and restored (Fig. 5a). The 3–5 rib models were prepared using 3D printing, and the titanium alloy rib locking plate was prefabricated accordingly.

During the surgeries, the 2 patients were placed in supine position after general anesthesia. As shown in Fig. 4b, a 6-cm chest incision was made, splitting to reveal the pectoralis major. Following the muscle fibers to avoid cutting across the muscle tissue until the ribs were exposed, and then following the surface of the ribs to expose the broken ends of the ribs. Due to the small incision and obvious deformation of the rib fracture, the MIPO system was performed to restore the broken ends of the rib fracture. Both ends of the fracture line were drilled with a MIPO right-angle drill and fixed with two screws. The two patients’ surgeries were successful, and the postoperative CT locking plate position and thoracic morphology were satisfactory (Figs. 4c, d, 5b, c).

### Post-operation

The 5 patients were routinely placed with 28F thoracic closed drainage tubes and negative pressure absorbing balls on the wound surface to expel pleural effusion and gas and facilitate pulmonary retraction and closure of the pleural cavity. There was no death reported in the whole cohort. The surgical time was 1–3 h and intraoperative blood loss was 100–150 ml. One patient was sent to ICU due to respiratory insufficiency after operation, and was successfully released after 1 day, then transferred back to the general ward. The negative pressure absorbing ball on the wound surface was pulled out within 48 h after operation. The chest drainage volume was less than 100 ml within 24 h. The chest film showed expansion after review and no obvious hydropneumothorax was found. Then the thoracic drainage tube was pulled out. Postoperative chest radiographs of each patient showed that the internal fixation had good and natural shape. There was good shape to the thoracic contour and was basically symmetrical with the contralateral side.

Ethical standards were followed in the content and dissemination of the study.

## Discussion

Rib fractures are most common in chest injuries, and the traditional treatment is mostly local compression bandaging, rib traction, selective mechanical ventilation and other methods [1]. Mechanical ventilation and prolonged hospital stay in the ICU were significantly associated with increased mortality and morbidity, particularly in elderly patients over 65 years of age [5]. The traditional internal fixation of rib fracture results in a large incision and a wide range of muscle cutting and fixation, which aggravates the trauma of patients. However, with continuous improvement and updating of medical devices and the trend toward minimally invasive surgery, the surgical treatment of multiple rib fractures has been accepted by more and more medical professionals [6, 7]. The thorax is a complex three-dimensional structure with different anatomical structures in different parts. The rib itself also has a relatively special and complex anatomical shape. In addition to the normal anatomic parameters such as length and width, the bending angle, longitudinal twist angle of each rib are important parameters that cannot be ignored, while the fixing devices that are currently most commonly used have undergone large improvements. However, it is still difficult to fully conform to the normal physiological and anatomical features of 3D bending and rib distortions.

**Fig. 6:**
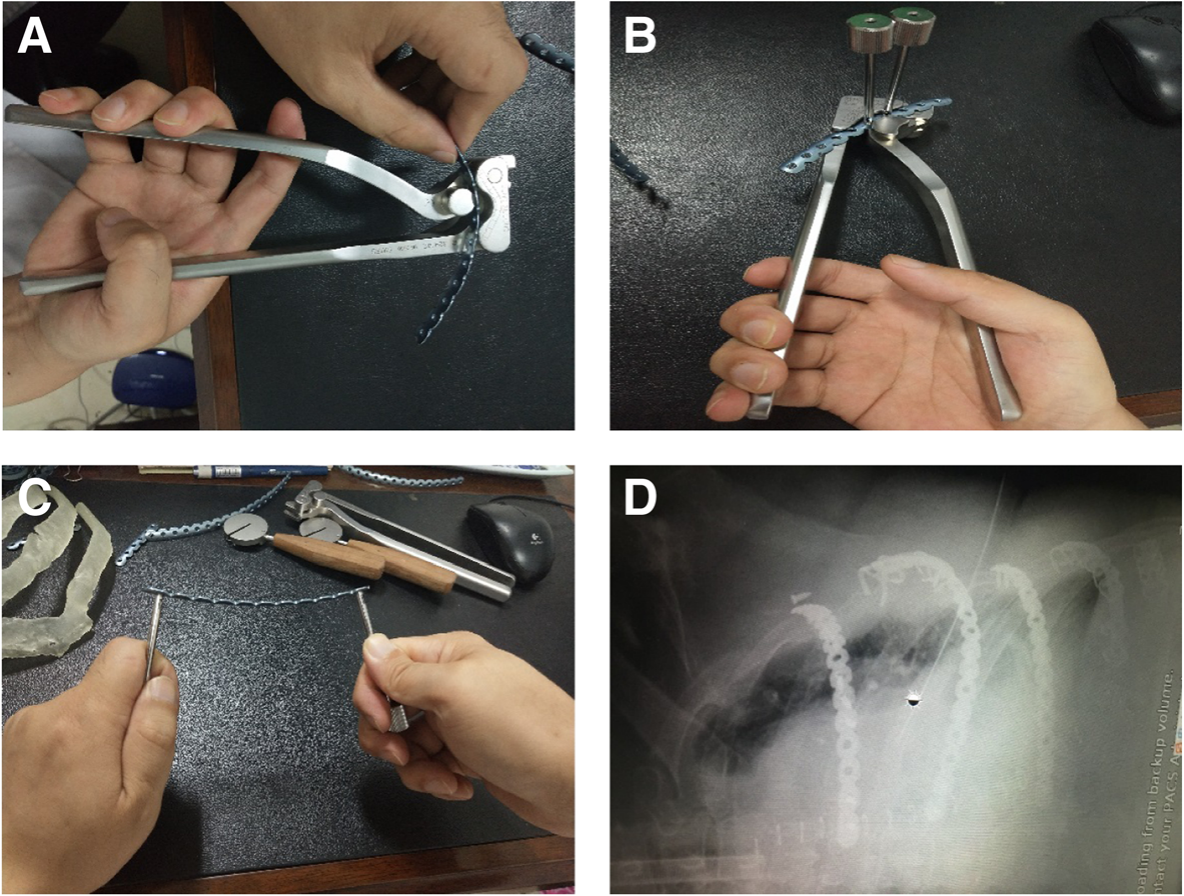
For fractures of the costal arch, a re-shaped locking plate is required. Adjust the (**a**) front and back radians of the rib lock, (**b**) upper and lower radians and (**c**) the rotation angle of the locking plate of the long axis of the ribs according to the 3D printing model. **d** the back end of the upper metal bone plate can be seen with abscission screw, the second metal bone plate is not ideal in shape, and the back end and the ribs are not well covered, and the embracing fixator is used as an accessory to prevent abscission

For severe multi-root and multi-segment rib fractures, as the rib has been severely deformed and the basis for fixation material is lost, there is a risk of incomplete thoracic molding for the re-shaped locking plate after the reduction of the rib during surgery [8]. In particular, the minimally invasive treatment of multiple fractures of multiple ribs in the area covered by the scapula and the pectoralis major (female breast), with the aid of the Matrix RIB system, is performed with the MIPO system tunnel [9, 10], because the operating space is relatively small [11] and it will be more difficult to restore the broken ends of each of the ribs and the plastic locking plate during surgery. For the fracture involving cartilage, especially the costal arch, the bending angle and longitudinal twisting angle of the ribs are more obvious [12]. There is no corresponding proprietary device for the current internal fixation materials, and the re-shaped locking plate is needed (Fig. 6a-c). Therefore, if the internal fixator is not in good shape and the two ends of the fracture are not a good fit, the internal fixator would produce twisting tension, while the rib is relatively weak, the fixation strength of the costal cartilage could be even worse [13]. This can easily lead to nail removal and the removal of the fixator after surgery (Fig. 6d).

3D printing is a methodology using three-dimensional CAD data sets for producing 3D haptic physical model [14]. In recent years, 3D printing technology has been applied in many medical fields to produce external prosthetics for cosmetic improvements and prefabricated molds for pre-surgical planning [15]. Furthermore, it can also utilized as implantable devices for shaft fractures of clavicles, acetabular fractures, and other surgical procedures. For example, Jeong HS et al. have reported that 3D printing technology for clavicles is very valuable for the accurate pre-bending plate with relative low cost, and the time to get the model is also short [16]. Studies have shown that surgeries combined with 3D printing technology help surgeons to understand anatomical and fracture morphology of patients, and shorten the operation time and reduce the radiation exposure time [17].

Based on the above problems, our hospital adopted 3D printing technology in the five cases. This method is more effective in term of keeping the thoracic integrity, and reducing the pulmonary complications. For cartilage fractures, especially for the costal arch fractures, the 3D printing technology is applied preoperatively to shape the locking plate according to the bending angle and longitudinal twisting angle of the ribs, which is more beneficial for fitting the fixator perfectly at both ends of the rib fracture and leads to a more effective recovery of thoracic integrity.

## Conclusion

In conclusion, for the 5 cases of multiple rib fractures reported in this study, the preoperative application of 3D printing technology can fully reduce the shaping time of intraoperative internal fixator, the difficulty of operation and the injury of patients. Therefore, for some specific types of rib fractures, the preoperative application of 3D printing technology has potential significance in achieving precise and individualized treatment, but this method still needs more clinical experience to provide better services for patients.

## Data Availability

All data presented are available from the corresponding author on reasonable request.

## Abbreviations

CT: Computed tomography
ICU: Intensive care unit
MIPO: Minimally invasive plate oseoynthesis
